# Influence of kilohertz frequency, burst duty cycle and burst duration on evoked torque, discomfort and muscle efficiency: A randomized crossover trial

**DOI:** 10.14814/phy2.70039

**Published:** 2024-10-16

**Authors:** Karenina Arrais Guida Modesto, Priscila Karen Silva Raposo, Isabella da Silva Almeida, Marco Aurélio Vaz, João Luiz Quagliotti Durigan

**Affiliations:** ^1^ Physical Education College, Program, Laboratory of Muscle and Tendon Plasticity University of Brasilia Brasília DF Brazil; ^2^ Faculty of Ceilândia, Laboratory of Muscle and Tendon Plasticity University of Brasilia Brasília DF Brazil; ^3^ Human Movement Sciences Program, ESEFID Federal University of Rio Grande Do Sul Porto Alegre RS Brazil; ^4^ Faculty of Ceilândia, Rehabilitation Sciences Program, Laboratory of Muscle and Tendon Plasticity University of Brasilia Brasília DF Brazil

**Keywords:** alternated current, burst‐modulated current, duty cycle, electrical stimulation, torque

## Abstract

Kilohertz‐frequency alternating currents (KFACs) have been indicated to minimize muscle atrophy and weakness. However, the optimal stimulation parameters still need to be determined.

**Objective:**

This study aimed to investigate the effects of different KFACs on evoked torque, current efficiency, and perceived discomfort.

**Design:**

KFACs with frequencies of 1 kHz (Aussie current) and 2.5 kHz (Russian current), along with two duty cycles (10% and 20%), were randomly applied to the triceps surae muscle of healthy participants using a crossover design. The NMES intensity, NMES‐evoked torque, NMES efficiency, and NMES discomfort were measured in maximal and submaximal conditions. Statistical analyses were conducted using a two‐way mixed‐model ANOVA with repeated measures. Forty‐four participants were included.

**Results:**

Aussie currents produced higher evoked torque and efficiency in maximal and submaximal efforts, with higher perceived discomfort in maximal effort. Although the Australian current may cause greater discomfort at maximal efforts, it matches the Russian current in perceived discomfort at submaximal levels. The 20% duty cycle produced the highest efficiency in submaximal efforts.

**Conclusion:**

In both maximal and submaximal efforts, the Aussie current demonstrated superior NMES efficiency, yielding higher torque with lower amplitude than the Russian current. Clinicians should take these findings into consideration when prescribing KFACs to optimize clinical outcomes.

## 1 INTRODUCTION

Kilohertz‐frequency alternating currents (KFACs) are a type of neuromuscular electrical stimulation (NMES) that gained attention in the 1970s after a Russian study demonstrated that these currents can lead to a gain in muscle strength (Ward & Shkuratova, 2002). It has been claimed that higher carrier frequencies in KFACs reduce skin impedance and allow less electrical energy dissipation, potentially generating greater evoked contractions (Da Silva et al., 2015; Medeiros et al., 2017). The Russian current was developed with parameters of 2.5 kHz, modulated in bursts of 50 Hz, with intervals of 10/10 ms burst/interburst and a burst cycle of 50% (Ward & Shkuratova, 2002). Since then, the studies by Ward et al. (2006); Ward and Lucas‐Toumbourou (2007), Ward and Robertson (1998a, 1998b) have shown that adjusting the carrier frequency to 1 kHz and using narrow burst durations, such as 2 and 4 ms (named the Aussie current), may offer distinct advantages in eliciting higher torque and reducing perceived discomfort compared to the Russian current.

KFACs present specific physical parameters, such as carrier frequency, pulse duration, burst duty cycle, and burst duration (Liebano, Rodrigues, et al., 2013; Liebano, Waszczuk, & Corrêa, 2013; Medeiros et al., 2017; Modesto et al., 2023; Pinto Damo et al., 2021; Ward et al., 2004, 2006). A recent systematic review investigated the relationship between the physical parameters of KFACs and the evoked torque, sensory discomfort, and muscle fatigue (Modesto et al., 2023). Carrier frequencies of up to 1 kHz were associated with higher discomfort, whereas carrier frequencies within the range of 2.5–5 kHz were associated with reduced discomfort, with one study indicating that a carrier frequency of 4 kHz induced lower discomfort when compared to lower carrier frequencies (Ward et al., 2004). When considering only torque, burst duty cycles shorter than 50% evoked greater torque, with the highest torque being evoked with a 20% burst duty cycle (Modesto et al., 2023). Although most studies have shown that shorter burst duty cycles (10%–50%) induced higher evoked torque and lower perceived discomfort (Liebano, Waszczuk, et al., 2013; Modesto et al., 2023; Parker et al., 2011; Szecsi & Fornusek, 2014; Ward et al., 2004), KFAC protocols have demonstrated inconsistent findings related to evoked torque and perceived discomfort and efficiency (Dantas et al., 2015; Modesto et al., 2023; Parker et al., 2005; Selkowitz et al., 2009; Ward et al., 2004, 2006) which may stem from non‐standardized NMES parameters, including carrier frequency, burst duration, and burst duty cycle (Modesto et al., 2023).

The evoked torque is significantly constrained by the magnitude of the current intensity, primarily due to the increased sensory discomfort levels with increasing current intensity (Pinto Damo et al., 2021), which have the potential to limit the NMES efficiency (Laufer & Elboim, 2008). A fundamental aspect to take into account is the NMES efficiency, which reflects the goal of maximizing the evoked torque output while minimizing the intensity of the applied NMES (Lieber & Kelly, 1991; Pinto Damo et al., 2021). In addition to assessing maximal NMES‐evoked torque, research has shown that dosages of submaximal evoked torque levels ranging from 5% to 50% are effective in strength training for different clinical populations. These findings highlight the importance of monitoring NMES efficiency at different submaximal intensity levels for muscle strengthening in clinical settings (Bellew et al., 2012; Dirks et al., 2015; Laufer & Snyder‐Mackler, 2010; Maffiuletti et al., 2011; Ward et al., 2002). For clinical applications, monitoring NMES efficiency at various submaximal intensity levels is crucial to optimize muscle‐strengthening protocols (Maffiuletti et al., 2011). This involves balancing evoked torque with tolerable discomfort levels to ensure patient compliance and the choice of KFAC current type (Modesto et al., 2023; Pinto Damo et al., 2021). However, no studies have yet simultaneously explored submaximal and maximal levels of both torque generation and perceived discomfort using various KFAC currents.

The primary aim of the current study was to compare the effects of two carrier frequencies in KFAC (2.5 kHz/Russian current and 1 kHz/Aussie current, with phase durations of 200 and 500 μs, respectively), applied at submaximal (20% of maximal voluntary isometric contraction—MVIC) and maximal tolerated current intensity levels, on NMES‐evoked torque, NMES efficiency, and discomfort in the triceps surae muscle of healthy participants. We also aimed to investigate the impact of altering the burst duty cycle (10% or 20%) with corresponding burst durations (2 or 4 ms). Our hypothesis was that using lower carrier frequencies and a shorter burst duty cycle would lead to increased evoked torque and improved current efficiency, at both maximal and submaximal levels, without significant differences in discomfort. Through these comparisons, the current study provides a snapshot of how the selection of NMES parameters for the two KFACs, based on muscle physiological assessment, may impact important outcomes for rehabilitation and training programs.

## 2 METHODS

### 2.1 Trial design

This study was a randomized, crossover, and double‐blind study. All participants provided their informed consent written prior to inclusion in the study. The study was approved by the Institutional Review Board at the University of Brasília (protocol 47989121600008093). The study is reported according to the Consolidated Standards of Reporting Trials (CONSORT, Supplementary Material). Registration: NCT05061056.

### 2.2 Participants

For inclusion, participants were required to be between 18 and 40 years old, physically active, and exhibit full range of motion in the knee joint, no prior experience with NMES, and no history of neuromuscular injury or disease. The sample size was determined a priori using G*Power (version 3.1.9.4) with the significance level set at *p* < 0.05, a power (1 − *β*) of 0.95, and an effect size of *f* = 0.42. Evoked torque values from a previous study (71.7 N. m ± 11.8, 76.9 N. m ± 14.1, 50.8 N. m ± 12.7, and 70.1.N.m ± 11), which followed a similar NMES protocol to that used in the present study, were used to calculate the effect size (Dantas et al., 2015). Thus, a sample of 44 participants was defined as the appropriate number of subjects, based on the calculation with the highest value among the primary outcomes (Bellew et al., 2021; Dantas et al., 2015; Paz et al., 2021), considering a sample loss of 20% (Bellew et al., 2021; Paz et al., 2021; Pinto Damo et al., 2021). Therefore, 44 participants were assessed for eligibility and included in the study (13 men and 31 women), age (mean ± SD) 25.65 ± 6.55 years, body mass 64.11 ± 11.95 kg, height 167.67 ± 0.09 cm, and body mass index 22.72 ± 3.30 kg⋅m^−2^.

### 2.3 Randomization and allocation concealment

Four NMES conditions were randomly presented: (1) 500 μs phase duration, low carrier frequency, and low burst duty cycle (1 kHz—AC 10% or Aussie current at 10% of burst duty cycle); (2) 500 μs phase duration, low carrier frequency, and high burst duty cycle (1 kHz—AC 20% or Aussie current at 20% of burst duty cycle); (3) 200 μs phase duration, high carrier frequency, and low burst duty cycle (2.5 kHz—RC 10% or Russian current at 10% of burst duty cycle); (4) 200 μs phase duration, high carrier frequency, and high burst duty cycle (2.5 kHz—RC 20% or Russian current at 20% of burst duty cycle; see Table 1 for further details). Computer‐generated randomization sequences were created using the website www.random.org, which assigned the stimulation protocols to each participant in a sequential manner. The order of NMES conditions for each participant was prepared by a single researcher (P.K.R.).

**TABLE 1:** Description of the physical parameters for the four types of electrical stimulation.

Current type	Carrier frequency (Hz)	Phase duration (μs)	Burst duration (ms)	Interburst duration (ms)	Burst duty cycle (%)	Burst frequency (Hz)
AC10%	1000	500	2	18	10	50
AC20%	1000	500	4	16	20	50
RC10%	2.500	200	2	18	10	50
RC20%	2.500	200	4	16	20	50

*Note*: All electrical currents had an on‐time of 6 s (1 s rise and 1 s of decline) and 60 s off‐time.

Abbreviations: AC, Australian current; RC, Russian current.

### 2.4 Blinding

The researcher (K.A.G.M.P) who applied the currents, and the participants were blinded to treatment allocation. To ensure blinding, a second researcher (P.K.R.) was responsible for programming each NMES and covering the device panel with a black cover to conceal parameters, except for current intensity, from both the researcher and participants.

### 2.5 Procedures

Participants were involved in five sessions, each of which lasted ~2 h, and separated by at least 7 days (Pinto Damo et al., 2021). A different type of NMES was tested in each session. Each participant was assessed in the same period of the day (at 2:00 or 6:00 pm) by the same assessor. Participants were instructed to avoid stimulants, such as alcohol, caffeine, and chocolate on testing days and not to engage in exercise before data collection. The first session served as a familiarization period during which subjects were informed about previous recommendations and study participation criteria. They also received an introduction to the testing procedures and protocols. Height, body mass, and BMI measurements were recorded for each participant.

All five experimental sessions were preceded by a warm‐up phase, during which two MVICs were performed to measure the highest ankle joint torque. Following the MVICs, a randomized NMES protocol was performed for that particular session, aiming to reach the maximal intensity tolerated by the participant (with the maximal evoked contraction, NMES‐MET, recorded) and the intensity that generated a submaximal evoked torque (NMES‐SET) with the NMES protocol (equivalent to 20% of MVIC). All procedures were conducted on the participants' right side. All NMES protocols were delivered with 50 Hz bursts (Table 1). To address potential clinical differences between KFAC currents, our study standardized carrier frequency, burst duration, and burst duty cycle parameters to provide a fair comparison. All currents were delivered with an “on” time of 8 s (1 s of ramp‐up, 6 s of constant time, and 1 s of decay) and an “off” time of 60s for recovery (Modesto et al., 2020).

### 2.6 Outcomes

The primary outcome was NMES‐evoked torque. Secondary outcomes were NMES efficiency and NMES discomfort.

### 2.7 Warm‐up and maximal voluntary isometric contraction

Participants sat in the chair of an Isometric Dynamometer (Cefise, Nova Odessa, SP) with their right foot secured to the footplate. Procedures were performed on the right leg, with the hip, knee, and ankle at ~90° (Grosprêtre et al., 2018). Subsequently, six submaximal isometric plantar flexion contractions, each lasting 6 s, were performed, with 10 s of rest between contractions. Following the warm‐up phase, the same position was maintained, and a 1‐min rest period was given. Two MVICs were then performed for 6 s each, with a 1‐min interval between attempts, and the highest torque achieved during these MVICs was recorded. MVIC torque was calculated at 6‐second window length.

### 2.8 NMES‐maximum evoked torque

Participants were instructed to relax the leg to avoid any voluntary contractions and were seated upright on the Isometric Dynamometer. Stimulation was produced using a neuromuscular electrical stimulator (version 2.0; Neurodyn, Ibramed, Amparo, SP, Brazil), connected to two pairs of 25 cm^2^ self‐adhesive electrodes (Valutrode, São Paulo, Brazil). The skin was shaved and cleansed with alcohol, and four electrodes were positioned on the triceps surae muscle group; two electrodes below the knee line (which forms the popliteal fossa) close to the proximal insertion of the medial and lateral gastrocnemius muscles, and two electrodes approximately 5 cm from the Achilles tendon, as previously described (Botter et al., 2011). Two minutes after completing the MVIC evaluation, the stimulation intensity was gradually increased from 10 mA, using 10 mA increments (Pinto Damo et al., 2021), until reaching 20% of the MVIC (SET). The intensity was then increased until the participant reported reaching the maximal tolerated intensity for that stimulus, determined in the familiarization session. The evoked torque was calculated at a 6‐s window length and was normalized (NMES‐evoked relative torque) using the following formula [(NMES‐evoked torque × voluntary torque^−1^) × 100] (Pinto Damo et al., 2021).

### 2.9 NMES‐efficiency assessment

NMES efficiency was calculated as NMES‐evoked torque/current intensity (Nm/mA) (Medeiros et al., 2017; Petrofsky et al., 2008; Pinto Damo et al., 2021).

### 2.10 Visual analogue scale

The Visual Analogue Scale (VAS) was used to assess sensory discomfort. This scale consists of a 10 cm line, where 0 cm represents no discomfort and 10 cm corresponds to the maximal discomfort perceived by the participant. The VAS was presented to the participants after the NMES‐induced contractions, during the familiarization sessions, and during all NMES‐evoked contractions.

### 2.11 Statistical analysis

The values for MVIC, evoked torque, muscle efficiency, and VAS were reported as mean and 95% confidence intervals. Parametric tests were used based on the normal distribution and homogenous variance (Levene's test) of the data. A two‐way mixed‐model ANOVA with repeated measures [two levels: currents × duty cycle (10% and 20%)] was utilized to compare the different groups for the afore mentioned outcomes. In case of a significant interaction effect, a Tukey post‐hoc test was used. All statistics were performed using the STATISTIC program, version 16. All graphs were designed with GraphPad Prism 6.0 software (San Diego, CA, USA). In addition, the effect size was calculated using Cohen's equation (Cohen, 1988). Effect sizes (*d*) were categorized as trivial (<0.20), small (0.20–0.49), moderate (0.50–0.79), large (0.80–1.29), and very large (>1.30) (Rosenthal, 1996). In addition, we calculated the Cohen's *f* effect size using G*Power software through the conversion of partial eta‐squared values provided by STATISTIC to Cohen *f* values. Cohen *f* values were interpreted as follows: small (0.10–0.24), medium (0.25–0.39), and large (*f* ≥ 0.40) effect (Cohen, 1988).

## 3 RESULTS

### 3.1 General evaluation

**FIGURE 1:**
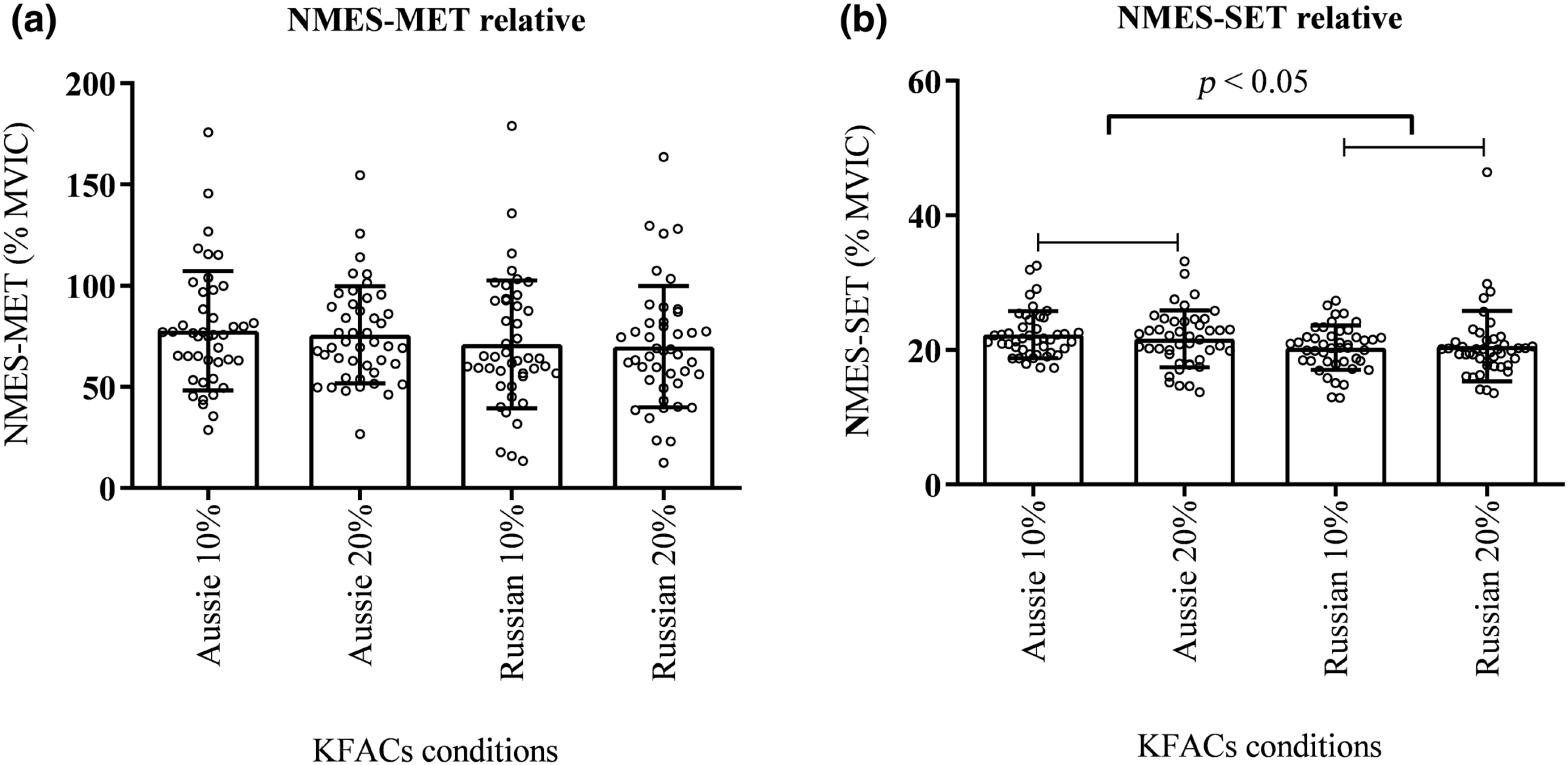
(a) There was no difference between neuromuscular electrical stimulation. (NMES) protocols for NMES‐maximum evoked torque (NMES‐MET) normalized by the maximal voluntary isometric contraction (MVIC). (b) Aussie currents evoked higher relative evoked torques when compared to Russian currents in submaximal evoked torque (NMES‐SET) in KFACs conditions.

There were no adverse effects during the application of the NMES protocols. All protocols presented a similar MVIC value (*p* = 0.225, *F* = 1.511, *η*_*ρ*_^2^: 0.033, power: 0.225; Table 2). There was no difference for the NMES‐MET relative torque (*p* = 0.893, *F* = 0.018, *η*_*ρ*_^2^: 0.000, power: 0.051; Figure 1a). For NMES‐SET, a main effect was found for current (*p* = 0.011, *F* = 6.930, *η*_*ρ*_^2^: 0.138; power: 0.730), where the Aussie current protocols presented a higher NMES‐SET relative torque when compared to the Russian current protocols (*p* < 0.05; Figure 1b).

**TABLE 2:** Results of different NMES protocols for maximum and submaximum torque.

	AC10%	AC20%	RC10%	RC20%
MVIC max (N. m)				
100%	76.70 (67.65–85.74)	76.55 (68.35–84.74)	71.74 (64.28–79.20)	76.39 (68.18–84.59)
Intensity (mA)				
20%	41.26^b^, ^c^ (37.85–44.67)	37.63^b^, ^c^ (34.63–40.63)	62.96^b^, ^c^ (57.95–67.97)	57.36^b^, ^c^ (51.89–62.83)
100%	71.00^a^ (63.57–78.42)	67.00^a^ (60.69–73.40)	92.95^a^ (86.11–99.79)	88.20^a^ (79.60–96.80)
MEC (N. m)				
20%	16.94^b^ (14.91–18.96)	16.34^b^ (14.52–18.16)	14.51^b^ (12.86–16.16)	15.30^b^ (13.74–16.85)
100%	56.37^a^ (49.81–62.92)	56.09^a^ (50.21–61.97)	46.90^a^ (41.91–54.36)	47.64^a^ (44.20–56.64)
Relative torque (%MVIC)				
20%	22.27^b^ (21.21–23.33)	21.65^b^ (20.37–22.93)	20.32^b^ (19.32–21.32)	20.58^b^ (18.98–22.18)
100%	77.68 (68.71–86.64)	75.74 (68.43–83.05)	69.96 (61.43–80.62)	67.64 (60.85–79.07)
NMES efficiency (Nm/mA)				
20%	43.87^b^, ^c^ (37.49–50.24)	46.03^b^, ^c^ (40.05–52.02)	24.28^b^, ^c^ (21.22–27.33)	29.20^b^, ^c^ (25.27–33.14)
100%	85.23^a^ (72.38–98.06)	86.99^a^ (77.81–96.16)	51.71^a^ (45.93–59.94)	57.80^a^ (52.25–68.12)
Discomfort (0–10)				
20%	3.42 (2.77–4.06)	3.39 (2.72–4.07)	3.72 (2.98–4.46)	3.78 (3.06–4.50)
100%	9.22^a^ (8.88–9.55)	9.27^a^ (8.98–9.57)	8.61^a^ (7.98–9.24)	9.07^a^ (8.77–9.37)

*Note*: Values are reported as mean (95% confidence interval).

Abbreviations: AC, Aussie currents; MEC, maximum evoked contraction; MVIC, maximum voluntary isometric contraction; NMES, neuromuscular electrical stimulation; RC, Russian currents.

^a^ Difference between collapsed Aussie current and collapsed Russian current for the maximum protocol.

^b^ Difference between collapsed Aussie current and collapsed Russian current for the submaximal protocol.

^c^ Difference between 10% duty cycle collapsed and 20% duty cycle collapsed for the submaximal protocol.

### 3.2 NMES‐maximum evoked torque

**FIGURE 2:**
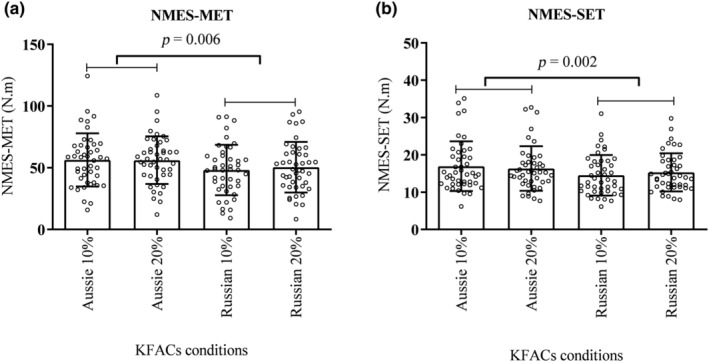
(a) Aussie currents evoked higher absolute neuromuscular electrical stimulation‐ maximum evoked torque (NMES‐MET) when compared to Russian currents in maximal in KFACs conditions. (b) Aussie currents presented a higher absolute NMES‐submaximal evoked torque (NMES‐SET) when compared to Russian currents in submaximal conditions.

No interaction was found between current and duty cycle (*p* = 0.564, *F* = 0.336, *η*_*ρ*_^2^: 0.007, power = 0.087). A main effect was found for the current (*p* = 0.006, *F* = 8.101, *η*_*ρ*_^2^: 0.158, power: 0.794), with the Aussie current conditions (AC 10% and AC 20%) presenting a higher NMES‐MET compared to the Russian current protocols (RC 10% and RC 20%) (*p* = 0.006) (Figure 2a), with large effect sizes (Table 3). No interaction was found between current and duty cycle for NMES_SET (*p* = 0.262, *F* = 1.29, *η*_*ρ*_^2^: 0.029, power = 0.199). However, a main effect was found for current (*p* = 0.002, *F* = 10.699, *η*_*ρ*_^2^: 0.199, power: 0.891), where the Aussie currents presented higher NMES‐SET compared to the Russian current protocols (*p* = 0.002) (Figure 2b), with large effect sizes (Table 3).

**TABLE 3:** Effect sizes.

Effect size, *d*							Among NMES currents		
	AC10% vs AC20%	AC10% vs RC10%	AC10% vs RC20%	AC20% vs RC10%	AC20% vs RC20%	RC10% vs RC20%	Partial eta squared	Effect size, *F*	Power
Intensity									
20% (currents)	0.34	1.53	1.07	1.86	1.36	0.32	0.71	1.59	1.00
20% (duty cycle)	0.34	1.53	1.07	1.86	1.36	0.32	0.13	0.40	0.72
100%	0.17	0.93	0.65	1.19	0.85	0.18	0.66	1.41	1.00
MEC									
20%	0.09	0.39	0.27	0.32	0.18	0.14	0.19	0.49	0.89
100%	0.01	0.39	0.28	0.39	0.28	0.11	0.15	0.43	0.79
Efficiency									
20% (currents)	0.10	1.19	0.84	1.39	1.01	0.42	0.59	1.21	1.00
20% (duty cycle)	0.10	1.19	0.84	1.39	1.01	0.42	0.12	0.37	0.67
100%	0.04	0.95	0.71	1.27	0.94	0.29	0.44	0.89	0.99
Discomfort									
20%	—	—	—	—	—	—	—	—	—
100%	0.04	0.36	0.14	0.40	0.20	0.28	0.13	0.38	0.70

*Note*: Effect sizes (*d*): trivial (<0.20); small (0.20–0.49); moderate (0.50–0.79); large (0.80–1.29); very large (>1.30). Effect size (*f*): small (0.10–0.24); medium (0.25–0.39); large (≥0.40).

Abbreviations: AC, Aussie currents; MEC, Maximum evoked contraction; NMES: Neuromuscular electrical stimulation; RC: Russian currents.

### 3.3 NMES efficiency

**FIGURE 3:**
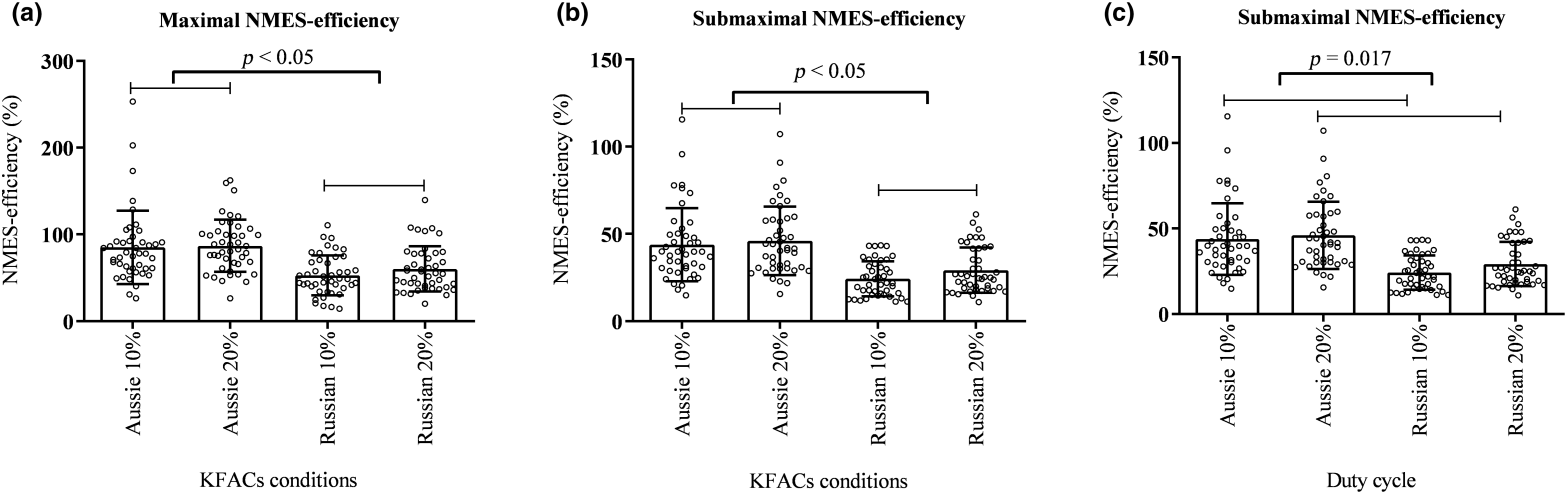
(a) Aussie currents presented higher neuromuscular electrical stimulation‐efficiency (NMES efficiency) when compared to Russian currents in maximal kilohertz‐frequency alternating currents (KFACs). (b) Aussie currents presented higher NMES efficiency when compared to Russian currents in submaximal conditions. (c) The 20% duty cycle presented higher NMES efficiency than the 10% duty cycle.

No interaction was observed in terms of efficiency between current and duty cycle for both maximal and submaximal levels (*p* = 0.497, *F* = 0.468, *η*_*ρ*_^2^: 0.010, power: 0.102; *p* = 0.458, *F* = 0.559, *η*_*ρ*_^2^: 0.012, power: 0.113, respectively). However, a main effect was found for both current conditions (*p* < 0.05, *F* = 35.175, *η*_*ρ*_^2^: 0.449, power: 0.999; *p* < 0.05, *F* = 63.115, *η*_*ρ*_^2^: 0.594, power: 1000, respectively). Aussie currents presented higher NMES efficiency compared to Russian currents (*p* < 0.001) (Figure 3a,b), with large effect sizes (Table 3). For submaximal NMES efficiency, a main effect for duty cycle was found (*p* = 0.017, *F* = 6.155, *η*_*ρ*_^2^: 0.125, power: 0.679). The 20% duty cycle showed greater NMES efficiency compared to the 10% duty cycle (*p* = 0.017; Figure 3c), with a medium effect size (Table 3).

### 3.4 NMES discomfort

**FIGURE 4:**
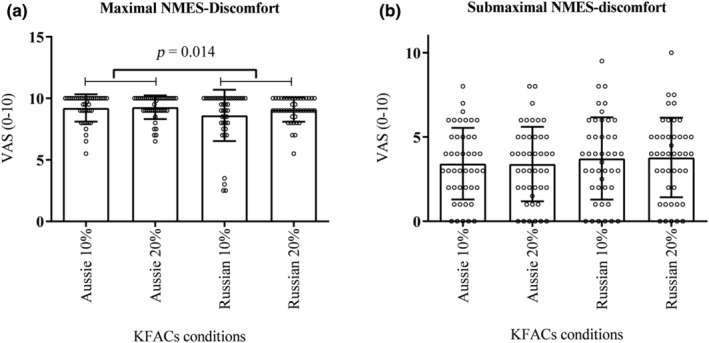
(a) Aussie currents presented higher neuromuscular electrical stimulation‐discomfort (NMES discomfort) when compared to Russian currents in maximal kilohertz‐frequency alternating currents (KFACs). (b) There was no difference between NMES protocols for submaximal discomfort.

For discomfort obtained at both maximal and submaximal levels, no interaction was found between current and duty cycle (*p* = 0.187, *F* = 1.794, *η*_*ρ*_^2^: 0.040, power: 0.258; *p* = 0.858, *F* = 0.032, *η*_*ρ*_^2^: 0.000, power: 0.053, respectively). For maximal discomfort, a main effect was found for current (*p* = 0.014, *F* = 6.496, *η*_*ρ*_^2^: 0.131, power: 0.702). Russian currents presented lower NMES discomfort compared to Aussie currents (*p* = 0.014; Figure 4a) with medium effect sizes (Table 3). There was no difference between NMES protocols for submaximal discomfort (*p* = 0.858, *F* = 0.032, *η*_*ρ*_^2^: 0.000, power: 0.053) (Figure 4b).

### 3.5 Intensity

**FIGURE 5:**
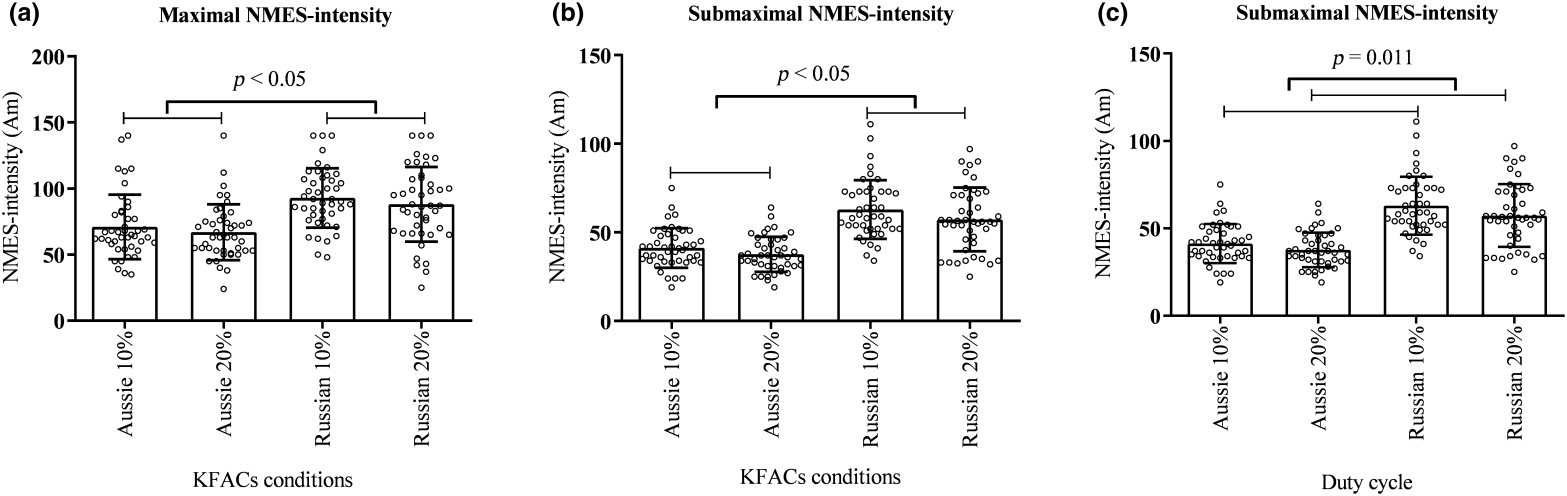
(a) Aussie currents presented lower generation of neuromuscular electrical stimulation intensity (NMES intensity) when compared to Russian currents in maximal kilohertz‐frequency alternating currents (KFACs). (b) Aussie currents presented lower generation of NMES intensity when compared to Russian currents in submaximal conditions. (c) The 20% duty cycle presented lower NMES intensity than the 10% duty cycle.

No interaction was found for current and duty cycle in relation to maximal and submaximal level intensities (*p* = 0.875, *F* = 0.025, *η*_*ρ*_^2^ < 0.001, power: 0.052; *p* = 0.055, *F* = 0.356, *η*_*ρ*_^2^: 0.008, power: 0.089, respectively). A main effect was found for current both for maximal and submaximal intensities (*p* < 0.05, *F* = 85.681, *η*_*ρ*_^2^: 0.665, power: 1.000; *p* < 0.05, *F* = 109.735, *η*_*ρ*_^2^: 0.718, power: 1.000, respectively). Russian conditions presented a higher NMES intensity compared to Aussie current conditions (*p* < 0.05) (Figure 5a,b) with large effect sizes (Table 3). There was a significant effect for duty cycle (*p* = 0.011, *F* = 6.911, *ηρ* ^2^: 0.138, power: 0.729), with the 20% duty cycle resulting in lower NMES intensity compared to the 10% duty cycle (*p* < 0.05; Figure 5c) for submaximal intensity. This difference between duty cycles showed large effect sizes (Table 3).

## 4 DISCUSSION

This was the first study to compare carrier frequency, burst duration, and burst duty cycle parameters by standardizing the stimulation parameters across different KFAC modalities widely used in clinical practice. These findings potentially suggest that the Aussie current can lead to higher levels of torque production compared to the Russian current, in both maximal and submaximal settings, regardless of the chosen duty cycle. Applying the Aussie current at maximal tolerated levels may lead to higher discomfort. Furthermore, our data indicate that the duty cycle does not appear to affect perceived discomfort across different KFACs. In clinical settings, the Aussie current emerges as a more efficient choice for both NMES‐MET and NMES‐SET production, while a 20% duty cycle demonstrates superior NMES efficiency, specifically for submaximal applications. This information is crucial for making evidence‐based decisions regarding NMES protocols in both exercise physiology and clinical settings.

The carrier frequency and burst duty cycle can impact both torque production and perception of discomfort during NMES. Choosing the right parameters has the potential to improve NMES‐ effectiveness (Modesto et al., 2023). Our results showed that the use of the Aussie current, with a lower carrier frequency of 1 kHz, led to greater evoked torque compared to the Russian current with a higher carrier frequency of 2.5 kHz. These results are consistent with previous studies, where higher torques were evoked with lower carrier frequencies (1 kHz), but these torques decreased significantly when frequencies exceeded 2.5 kHz (Ward et al., 2004, 2006). It appears that the 1 kHz carrier frequency is the most effective among the KFACs in terms of evoking the highest torque, as it also produced a higher evoked torque compared to KFAC at 4 kHz (Medeiros et al., 2017). However, above 2.5 kHz, it appears that the evoked torque is similar among KFACs with higher carrier frequencies, as shown when 2.5 kHz was compared to 5 kHz (Selkowitz et al., 2009).

We compared the Aussie current, with a lower carrier frequency (1 kHz) and longer pulse duration (500 μs), to the Russian current, with a higher carrier frequency (2.5 kHz) and shorter pulse duration (200 μs). This comparison was selected because pulse duration seems to have a significant effect on the neuromuscular system response (Medeiros et al., 2017). The intensity/duration curve (I/T curve) (Robertson et al., 2006) suggests that with long‐duration pulses, the thresholds for large and small‐diameter fibers are closer together. This indicates that a wide range of fiber diameters will be stimulated with supra‐threshold stimulation. The motor neurons innervating skeletal muscles have a variety of fiber diameters, thus it would be expected that a larger population of muscle fibers would be recruited with long‐duration pulses (Li & Bak, 1976; Ward et al., 2004). Research has indicated that wide pulses generate greater torque, which aligns with the idea that total electrical charge (i.e., the product from current intensity times pulse duration) plays an important role in NMES‐induced torque production (Medeiros et al., 2017). These findings suggest that the variations in torque production associated with KFAC carrier frequencies may not only be attributed to the carrier frequencies. It appears that pulse duration also plays a significant role, as there is an inverse relationship between pulse duration and carrier frequency. Furthermore, an increase in pulse duration reduces the current intensity required to attain the motor threshold (Modesto et al., 2023). Our findings indicate that opting for a lower KFAC carrier frequency results in longer pulse durations, which in turn leads to increased torque generation, while simultaneously reducing current intensity and maximizing NMES effectiveness.

In clinical practice, the amount of evoked torque plays a crucial role for inducing strength gains and preventing muscle wasting among different patient populations. The recommended NMES‐evoked torque has been suggested to range between 5% and 50% of MVIC (Gibson et al., 1988; Laufer & Snyder‐Mackler, 2010; Snyder‐Mackler et al., 1994). In the current study, the maximal current intensity tolerated by participants evoked a relative torque of ~66% MVIC across all NMES conditions. However, our data showed that the Aussie current applied at the maximal tolerated current intensity may lead to increased discomfort compared to submaximal torque settings where no significant between current difference in discomfort was observed. This result indicates than when prioritizing NMES comfort, clinicians may prefer to consider using the Aussie current at a submaximal evoked effort level of 20% MVIC. This threshold falls within the range considered effective for strengthening in various patient populations (Dirks et al., 2015; Gibson et al., 1988; Laufer & Snyder‐Mackler, 2010; Maddocks et al., 2016; Snyder‐Mackler et al., 1994).

The duty cycle is another factor that, along with the carrier frequency, has been suggested to influence torque production evoked by KFAC (Modesto et al., 2023). However, the current study found no significant differences in evoked torque between different duty cycles using two types of KFAC. This finding is in agreement with previous studies that showed a greater evoked torque when using burst durations of 2–4 ms and shorter duty cycles (Liebano, Waszczuk, et al., 2013; McLoda & Carmack, 2000; Parker et al., 2011; Szecsi & Fornusek, 2014; Ward et al., 2004), which were also applied in the present study. McLoda and Carmack (2000) compared duty cycles ranging from 10% to 90% and observed that the average evoked force decreased as the duty cycle increased, with the 10% duty cycle producing greater force than the 90% duty cycle. Furthermore, the average efficiency was higher with a 10% duty cycle compared to a 90% duty cycle. Ward et al. (2004) tested different duty cycles, ranging from 0% to 100%, using different carrier frequencies, and found that duty cycles lower than 20% resulted in greater evoked torque, regardless of the carrier frequency. The similarity in the duty cycles used in this study (10% and 20%) may explain the absence of significant differences when comparing our data to the studies conducted by McLoda and Carmack (2000) and by Ward et al. (2004). Nevertheless, our results indicate that using a 20% duty cycle provides greater NMES efficiency. Given that submaximal evoked (20% of MVC) applications are thought to rely on large afferent volleys being sent to the spinal cord, (Maddocks et al., 2016) the present results suggest that a 20% duty cycle applied at submaximal evoked torques may produce the largest afferent volley, influencing important outcomes for rehabilitation and training programs.

The perceived discomfort can be a limiting factor in the efficiency of NMES application (Bax et al., 2005; Maffiuletti et al., 2011; Vaz & Frasson, 2018). Previous studies have shown that carrier frequencies of 2.5, 5, and 10 kHz resulted in less perceived discomfort (Rooney et al., 1992). In our study, no differences between currents (1 kHz vs. 2.5 kHz) were observed when generating submaximal torques, possibly due to the proximity of the carrier frequency values. In contrast, the 1 kHz carrier frequency generated greater discomfort during NMES‐MET. Lower carrier frequencies result in an increased pulse duration, so that the NMES achieves the motor and pain thresholds more easily, thereby increasing discomfort (Ward & Chuen, 2009). In contrast, a study conducted by Medeiros et al. (2017) reported no difference in perceived discomfort between carrier frequencies of 1 and 4 kHz when producing maximal torques. Individual variability, influenced by past negative experiences with NMES, fear, apprehension, and anxiety levels, can also contribute to the emotional dimension of perceived discomfort (Delitto et al., 1992; Medeiros et al., 2017).

In addition, factors such as the duty cycle and neural firing rate can have a significant impact on discomfort perception. For instance, when the duty cycle is set at 50%, resulting in a firing rate of less than 500 Hz, it can lead to rapid fiber inactivation due to neurotransmitter depletion or nerve block (Bowman & McNeal, 1986; Jones, 1996; Ward & Robertson, 2001). On the other hand, lower duty cycles of around 20%, with a firing rate under 200 Hz, may mitigate this by avoiding excessive rates that cause propagation failure or sensory overload within the nerves (Modesto et al., 2023). Szecsi and Fornusek (2014) found that a 50% duty cycle was more uncomfortable compared to duty cycles below 50% (2%–50%). However, in the present study, differences were observed between duty cycles of 10% and 20% only in maximal conditions. It has been suggested that optimal blocking of pain fiber activity occurs when sensory fibers are stimulated at firing rates of around 100 Hz (Johnson et al., 1989; Selkowitz et al., 2009). Interestingly, for maximal efforts, the Aussie current was found to be more uncomfortable, which might be due to individual emotional factors, such as apprehension about experiencing maximal discomfort when using the highest tolerated intensity (Medeiros et al., 2017). Collectively, the conflicting results in perceived discomfort highlight the need for standardized evaluation methods that consider both the physical and emotional contexts influencing individual experiences during NMES in clinical settings.

## 5 LIMITATIONS

The present study acknowledges some limitations. Our assessment was limited to healthy young participants with relatively low body mass index values, which may not fully represent clinical populations and athletes. Our sample included both men and women, reflecting the typical scenario found in clinical practice and reinforcing the external validity of the study. We did not control for any NMES outcomes related to sex differences between participants, which could be a limitation regarding perceived discomfort (Medeiros et al., 2017). Additionally, since our study primarily focused on the acute effects of NMES currents, future studies should be conducted to investigate the chronic effects of KFACs related to NMES‐evoked torque, discomfort, and efficiency across different carrier frequencies and burst duty cycles in both healthy and clinical populations.

## 6 CONCLUSION

Our study demonstrated that the Ausssie current (1 kHz) outperforms the Russian current (2.5 kHz) in terms of evoked torque and efficiency, both in maximal and submaximal conditions. While the Aussie current may lead to greater discomfort in maximal conditions, it matches the Russian current in submaximal conditions for perceived discomfort. Lastly, employing a 20% duty cycle enhances efficiency and reduces the required current intensity during submaximal conditions. Clinicians should consider the carrier frequency, along with the duty cycle, when recommending KFAC for optimal NMES efficiency in clinical practice.

## AUTHOR CONTRIBUTIONS

Authors Karenina Arrais Guida and Priscilla Karen Silva Raposo carried out the data collection in collaboration with author Isabella da Silva Almeida. Authors Karenina Arrais Guida Modesto, Marco Aurélio Vaz and João Durigan carried out the statistical analysis. All authors participated in writing the manuscript.

## FUNDING INFORMATION

There was no funding for this study.

## CONFLICT OF INTEREST STATEMENT

The authors declare that the research was conducted in the absence of any commercial or financial relationships that could be construed as a potential conflict of interest.

## ETHICS STATEMENT

We declared that this study was approved by the Institutional Review Board at the University of Brasília (protocol 47989121600008093).

## Supporting information


Data S1.

## Data Availability

Data will be made available upon reasonable request.
